# The design of trapping devices in pollination traps of the genus *Arum* (Araceae) is related to insect type

**DOI:** 10.1111/boj.12054

**Published:** 2013-06-03

**Authors:** David Bröderbauer, Anton Weber, Anita Diaz

**Affiliations:** 1 Department of Structural and Functional Botany, University of ViennaRennweg 14, 1030, Vienna, Austria; 2 School of Applied Sciences, Bournemouth UniversityDorset House, Talbot Campus, Fern-Barrow, Poole, BH12 5BB, UK

**Keywords:** deceptive pollination, epidermis, floral traits, morphology, natural selection

## Abstract

Pollinators have long been known to select for floral traits, but the nature of this relationship has been little investigated in trap pollination systems. We investigated the trapping devices of 15 *Arum* spp. and compared them with the types of insects trapped. Most species shared a similar general design of trap chamber walls covered in downward-pointing papillate cells, lacunose cells in the chamber wall and elongated sterile flowers partially blocking the exit of the trap. However, there was significant variation in all these morphological features between species. Furthermore, these differences related to the type of pollinator trapped. Most strikingly, species pollinated by midges had a slippery epidermal surface consisting of smaller papillae than in species pollinated by other insects. Midge-pollinated species also had more elongated sterile flowers and tended to have a larger lacunose area. We conclude that pollination traps evolve in response to the type of insect trapped and that changes to the slippery surfaces of the chamber wall are an important and previously little recognized variable in the design of pollination traps. © 2013 The Linnean Society of London, *Botanical Journal of the Linnean Society*, 2013, **172**, 385–397.

## Introduction

It is well known that pollinators select for floral traits in flowers and thus affect the size and shape of floral organs (e.g. Schemske & Bradshaw, 1999; Sletvold *et al*., 2012). In rewarding flowers, stabilizing selection on advertisement traits is thought to ensure recognition by pollinators that learn to remember floral characters associated with the reward (Ackerman, Cuevas & Hof, 2011). In contrast, in deceptive flowers variability in floral traits is presumed to be higher so that pollinators are not able to learn to discriminate between the deceptive flower and the imitated rewarding model (Ayasse *et al*., 2000).

Some deceptive flowers not only mimic rewards but also trap their pollinators in order to ensure pollen transfer (Vogel & Martens, 2000). Such pollination traps have evolved in various angiosperm lineages. They are characterized by a chamber formed by tepals or modified bracts that enclose the flowers (Vogel, 1965). Different morphological adaptations enable the trapping of the insect pollinators inside the chamber. For example, slippery surfaces covering the chamber walls occur in several clades (Poppinga *et al*., 2010). These surfaces usually consist of downward-pointing papillate cells or an epicuticular wax layer that disable the attachment organs of the insect and cause it to slip into the floral chamber (Gaume *et al*., 2004). Some pollination traps have hairs on the chamber walls that block the exit of the floral chamber (Oelschlägel *et al*., 2009). In some taxa, the entire floral chamber becomes temporarily occluded by a constriction of the chamber wall (Ulrich *et al*., 2012). Insects can escape from the floral chamber only after pollen release, when the exit reopens and/or after the trapping devices have wilted (Bröderbauer, Diaz & Weber, 2012). However, the extent to which these trapping devices are under selection based on the type of pollinator caught is currently an unexplored question.

**Figure 1:**
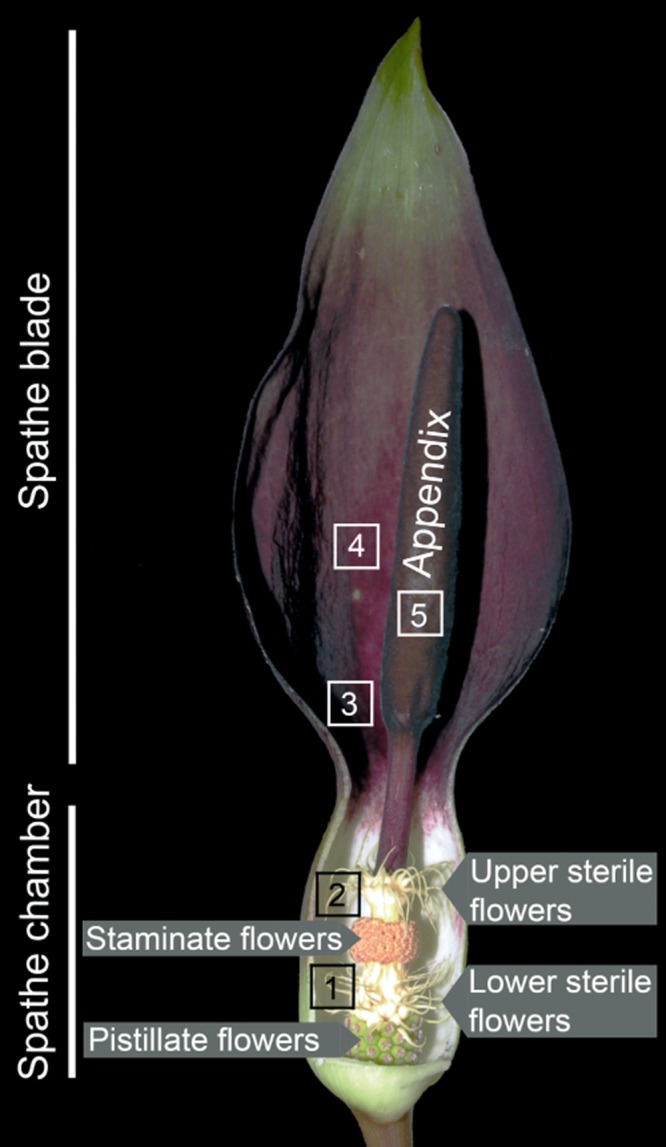
Inflorescence of *Arum elongatum* consisting of the flower-bearing spadix and the surrounding spathe. The numbers in boxes indicate the parts of the spathe from which samples were taken (1, lower spathe chamber; 2, upper spathe chamber; 3, spathe blade 1 cm above the spathe constriction; 4, central spathe blade; 5, central part of the appendix). The frontal part of spathe chamber has been removed to show the flowers inside.

The genus *Arum* L. (Araceae) offers an excellent opportunity to explore the relationship between pollinators and floral structure as it comprises 29 species (Boyce, 1993; Linz *et al*., 2010), which attract various types of pollinators (reviewed in Gibernau, Macquart & Przetak, 2004). All *Arum* spp. have highly synorganized inflorescences consisting of a flower-bearing spadix (with pistillate, staminate and sterile flowers) that is surrounded by a modified bract, the spathe (Fig. 1). The adaxial spathe epidermis consists of downward-pointing papillate cells that are slippery and cause the insects to glide into the floral chamber (Knoll, 1926), which is formed by the inflated spathe. Intercellular spaces in the wall of the spathe chamber, called lacunae, are assumed to support the oxygen supply, which is believed to prevent the suffocation of pollinators during their arrest (Knoll, 1923; Bermadinger-Stabentheiner & Stabentheiner, 1995). Elongated sterile flowers situated on the spadix below the appendix and below the staminate flowers (Fig. 1) are also slippery and hinder the escape of the trapped insects (Knoll, 1926). Papillate cells are also found on the sterile appendix that sits on top of the staminate flowers (Fig. 1) and produces heat and odour (Mayo, Bogner & Boyce, 1997). In the majority of species studied so far, the pollinators are trapped in the floral chamber for *c*. 24 h (e.g. Diaz & Kite, 2002; Quilichini *et al*., 2010; Stökl *et al*., 2010). The pollinators include saprophilous flies and beetles, midges and bees (e.g. Gibernau *et al*., 2004).

The overall aim of the present study is to compare the relationship between trapping devices and types of pollinators in *Arum* spp. The specific questions are: (1) how variable is the overall design of the trapping inflorescences within the genus *Arum*?; (2) are differences in the design of the respective trapping devices related to differences in the type of insects trapped?

## Material and Methods

### Comparison of trap design

To compare the trap design of the *Arum* spp. examined, we investigated all inflorescence structures that contribute to the trapping of the pollinators. These are (1) the papillate epidermal cells that form the slippery surface and cover the spathe and the spadix, (2) the lacunae in the spathe tissue of the spathe chamber and (3) trapping hairs, i.e. the elongated sterile flowers situated below and above the staminate flowers. Despite best efforts no more than 15 of the 29 *Arum* spp. were available for our study. These species cover the majority of the clades in the genus (Espíndola *et al*., 2010) and represent all pollination syndromes found in *Arum* (Linz *et al*., 2010). Voucher specimens are preserved in spirit collections of the Herbarium of the University of Vienna (WU) (see Table 1).

**Table 1:** Voucher specimens of *Arum* spp. deposited in the herbarium of the University of Vienna (WU) and the private spirit collection of the first author (BRO)

*A. balansanum* R.R.Mill, WU 0064937. *A. besserianum* Schott, WU 0064939. *A. concinnatum* Schott, BRO 11092012. *A. creticum* Boiss. & Heldr., WU 0064940. *A. cylindraceum* Gasp., WU 0064941. *A. dioscoridis* Sm., WU 0064942. *A. elongatum* Steven, WU 0064943. *A. euxinum* R.R.Mill, WU 0064946. *A. hygrophilum* Boiss., BRO 11092016. *A. idaeum* Coustur. & Gand., WU 0064945. *A. italicum* Mill., WU 0064947. *A. maculatum* L., BRO 11092014. *A. megobrebi* Lobin, M.Neumann, Bogner & P.C.Boyce, WU 0064948. *A. nigrum* Schott, WU 0064949. *A. purpureospathum* P.C.Boyce, WU 0064950.

### Relationship between pollinators and trapping devices

Information on pollinators was taken from the literature (Table 2). We grouped *Arum* spp. according to the composition of their pollinating fauna as follows: (1) bees (Hymenoptera); (2) flies and beetles (Diptera-Brachycera and Coleoptera); (3) midges (Diptera–Nematocera). To compare *Arum* spp. in terms of their papillate epidermal cell slippery surfaces and lacunae, inflorescences were collected during anthesis and were preserved in 70% alcohol. For investigation under scanning electron microscopy (SEM) samples were taken from five different regions of the spathe: (1) lower spathe chamber at the level of the lower sterile flowers; (2) upper spathe chamber at the level of the upper sterile flowers; (3) 1 cm above the spathe constriction; (4) from the central part of the spathe blade; and (5) from the central part of the spadix–appendix (Fig. 1). Samples were dehydrated in a graduated series of ethanol and then infiltrated with acetone. Afterwards samples were critical-point-dried, sputter-coated with gold and investigated with a JEOL JSM6390 SEM.

**Table 2:** Species of *Arum* investigated and their pollinators. Note: taxa in brackets represent visitors that are most likely not involved in pollination according to the more recent literature

Species	Pollinator type	Taxa	Source
*A. balansanum* R.R.Mill	Unknown	Unknown	
*A. besserianum* Schott	Unknown	Unknown	
*A. concinnatum* Schott	Flies and beetles	Chironomidae, Drosophilidae, Psychodidae, Sciaridae, Sphaeroceridae, Staphylinidae	Drummond & Hammond, 1993; Urru *et al*., 2010
*A. creticum* Boiss. & Heldr.	Bees	Halictidae (Miridae, Chrysomelidae, Melyridae, Scarabeaidae)	Diaz & Kite, 2006 (Drummond & Hammond, 1993)
*A. cylindraceum* Gasp.	Midges	Culicidae, Psychodidae	Gibernau *et al*., 2004, Revel *et al*., 2012
*A. dioscoridis* Sm.	Flies and beetles	Scarabaeidae, Sphaeroceridae, Staphylinidae	Kullenberg, 1953; Papp & Rohacek, 1987; Drummond & Hammond, 1991
*A. elongatum* Steven	Midges	Ceratopogonidae	Braverman & Koach, 1982; Koach, 1985
*A. euxinum* R.R.Mill	Midges	Psychodidae, Sphaeroceridae	Gibernau *et al*., 2004; Linz *et al*., 2010
*A. hygrophilum* Boiss.	Midges	Psychodidae	Koach, 1985; Gibernau *et al*., 2004
*A. idaeum* Coustur. & Gand.	Bees	Halictidae (Miridae, Chrysomelidae, Melyridae, Mordellidae)	Diaz & Kite, 2006
*A. italicum* Mill.	Midges	Chironomidae, Psychodidae, Ceratopogonidae, Drosophilidae	Diaz & Kite, 2002; Albre, Quilichini & Gibernau, 2003; Gibernau *et al*., 2004
*A. maculatum* L.	Midges	Psychodidae	Rohacek, Beck-Haug & Dobat, 1990; Lack & Diaz, 1991; Diaz & Kite, 2002; Gibernau *et al*., 2004, Espíndola & Alvarez, 2011
*A. megobrebi* Lobin, M.Neumann, Bogner & P.C.Boyce	Unknown	Unknown	
*A. nigrum* Schott	Flies and beetles	Sphaeroceridae, Staphylinidae	Knoll, 1926; Gibernau *et al*., 2004
*A. purpureospathum* P.C.Boyce	Unknown	Unknown	

The nature of the slippery surfaces of each species was measured in terms of the basal area of papillae (*N* = 10) and the length of papillae (*N* = 10) for the upper spathe chamber, the lower and the central spathe blade and the spadix–appendix. As the lower spathe chamber was found not to contain trapping devices, it was excluded from further statistical analyses. The average area of the upper spathe chamber covered with lacunae (*N* = 10) was measured by multiplying the average lacuna size (*N* = 10) by the number of lacunae given for an area under 500 μm magnification. The elongated sterile flowers of each *Arum* sp. were compared simply in terms of the number of elongated sterile flowers found below and above the staminate flowers. These data were not taken from the same individual plants as the data for papillate surfaces and lacunae and so were analysed separately.

Univariate analyses were carried out using SPSS version 15. We tested for differences in the design of the papillate cell slippery surfaces, lacunae and sterile flowers between all 15 species and between the pollinator types using Kruskal–Wallis. Post hoc Mann–Whitney *U*-tests were carried out on each pair of groups applying a Bonferroni correction using PAST (Paleontological Statistics) version 2.17 (Natural History Museum University of Oslo 1999–2012). For the Kruskal–Wallis analysis, the four species with unknown pollinators were removed as they could not be assigned to any group. Multivariate analyses were carried out using Primer version 6 (Clarke & Gorley, 2006). Firstly, we gained a visual representation of the combined differences in plant pollination traits between species by applying a non-metric multidimensional scaling (NMDS) analysis based on the Bray–Curtis similarity index with no data transformation and no normalization. The stress value calculated in the NMDS is an estimate for the adequacy of the NMDS representation indicating the goodness of fit (Clarke & Warwick, 2001). Stress values below 0.1 correspond to a good ordination with no real prospect of a misleading interpretation.

Then, in order to test whether there were significant differences between plant species and insect groups, we conducted a one-way analysis of similarity (ANOSIM) with 999 permutations. The global R statistic that results from ANOSIM represents similarity and generally ranges from 0 (total similarity) to 1 (total dissimilarity). As the measures for the area and the length of papillae could not be taken at one time for the same papilla during investigation under SEM, we had to combine data on length and area originating from different papillae. Therefore, the data for a single point in the NMDS stem from different papillae, whereby every point becomes a pseudo-individual. Nevertheless, our results indicate that the data are representative, as the pseudo-individuals of the respective species always grouped together.

## Results

### Comparison of trap design

**Figure 2:**
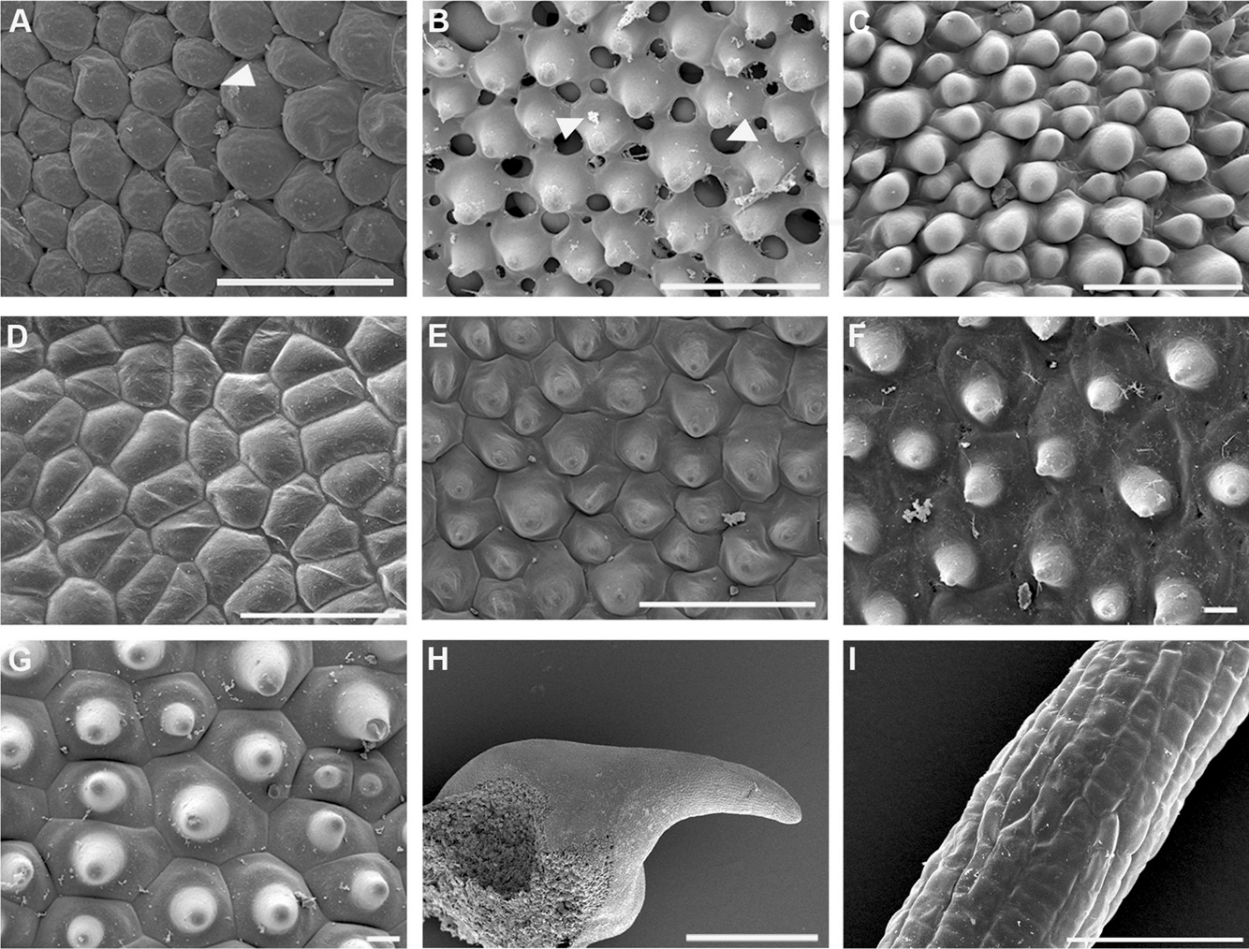
Trapping devices in *Arum*. A, *A. megobrebi*, lower spathe chamber, convex epidermal cells. Note the small lacunae in the cell corners (arrowhead); B, *A. besserianum*, upper spathe chamber, downward-pointing papillate epidermal cells with lacunae in the cell corners (arrowheads); C, *A. creticum*, upper spathe chamber, downward-pointing papillate epidermal cells. Note that lacunae are absent; D, *A. idaeum*, spathe blade, tabular epidermal cells; E, *A. concinnatum*, spathe blade, downward-pointing papillate epidermal cells; F, *A. italicum*, spathe blade, downward-pointing papillae emerging from tabular epidermal cells; G, *A. euxinum*, appendix, straight papillae; H, *A. nigrum*, elongated sterile flower; I, *A. cylindraceum*, tabular epidermis of the elongated sterile flower. Scale bars, 1000 μm (K); 100 μm (A–E, H–I); 10 μm (F–G).

Apart from *A. creticum* Boiss. & Heldr. and *A. idaeum* Coustur. & Gand., spathes of all *Arum* spp. studied showed a clear zonation and a consistent set of features. The lower spathe chamber consisted of unspecialized tabular to convex epidermal cells, often with small intercellular spaces in the cell corners (Fig. 2A). In the upper part of the spathe, chamber cells were papillate and downward-pointing and lacunae (i.e. large intercellulars) occurred in the corners of the papillate cells (Fig. 2B). In *A. creticum* and *A. idaeum*, the spathe chamber lacked lacunae (Fig. 2C). Moreover, in *A. idaeum* papillae were absent in the entire spathe (Fig. 2D). In the other species, the epidermis of the spathe blade was made up of downward-pointing papillae and lacunar tissue was absent (Fig. 2E). In some species the papilla covered the whole cell surface (Fig. 2E) (*A. concinnatum* Schott, *A. creticum*, *A. dioscoridis* Sibth. & Sm. and *A. nigrum* Schott), but in the other species the papillae emerged from tabular cell surfaces (Fig. 2F). The appendix was covered with papillae in all species except for *A. idaeum*. However, in contrast to the downward-pointing papillae covering the spathe, the papillae on the appendix were perpendicular to the surface or only slightly downward-pointing (Fig. 2G). The epidermis of the elongated sterile flowers was tabular (Fig. 2H, I). In all species, stomata were rare on the adaxial (i.e. inner) spathe epidermis, but common on the abaxial epidermis, especially along the spathe chamber.

### Relationship between pollinators and trapping devices

The means ± standard deviation (SD) of measurements of papillate cell slippery surfaces are shown in Table 3. In general, species pollinated by flies and beetles had longer papillae with a larger basal area than those pollinated by midges, whereas the lacunose area was larger in the latter group. However, *A. concinnatum* (purportedly pollinated by flies and beetles) showed some deviations. Its papillae, although longer than in most species pollinated by midges, were still shorter than in *A. maculatum* L. (midges). Moreover, the lacunose area of *A. concinnatum* was much larger than in the other two species pollinated by flies and beetles (*A. dioscoridis* and *A. nigrum*). The two species pollinated by bees had no lacunae and lacked elongated sterile flowers. Papillae were absent in *A. idaeum*, whereas papillae of *A. creticum* appeared to be intermediate in length between those found in the group of species pollinated by flies and beetles and the group pollinated by midges. Regarding basal area, the papillae of *A. creticum* were much broader than those of midge-pollinated species and were in most parts of the epidermal surface of similar basal area as those of fly and beetle pollinated species. In general, the number of elongated sterile flowers tended to be greater in species pollinated by midges than in species pollinated by flies and beetles, with the exception of *A. concinnatum* (flies and beetles), which had that highest number of sterile flowers out of the l3 species measured (Table 2), and *A. hygrophilum* Boiss. (midges), which had a low number of sterile flowers. All structures of the slippery surfaces and the area covered by lacunae differed significantly between the species compared and these differences persisted when species were grouped according to their pollinator type (Table 4).

**Table 3:** Measures of trapping devices in *Arum* spp

Species	Length of papilla S2 (μm)	Length of papilla S3 (μm)	Length of papilla S4 (μm)	Length of papilla S5 (μm)	Basal area of papilla S2 (μm^2^)	Basal area of papilla S3 (μm^2^)	Basal area of papilla S4 (μm^2^)	Basal area of papilla S5 (μm^2^)	Area of lacunae S2 (μm^2^)	Number of upper sterile flowers (*N*)	Number of lower sterile flowers (*N*)
*A. creticum* (bees)	41.90 ± 5.36	28.00 ± 3.68	29.90 ± 3.98	19.40 ± 4.14	527.00 ± 63.56	681.20 ± 225.74	679.30 ± 161.50	708.20 ± 207.85	0	1.1 (10) ± 1.9	0.5 (10) ± 1.3
*A. idaeum* (bees)	0	0	0	0	1046.10 ± 278.67	964.50 ± 162.65	1452.40 ± 289.79	898.50 ± 198.28	0	0.0 (10) ± 0	0.0 (10) ± 0
*A. concinnatum* (flies and beetles)	31.00 ± 2.11	24.70 ± 4.32	22.80 ± 4.08	25.00 ± 5.27	669.60 ± 122.38	598.40 ± 221.61	625.40 ± 108.99	553.90 ± 165.28	7782.21 ± 743.35	87.4 (5) ± 8.6	20.6 (5) ± 6.5
*A. dioscoridis* (flies and beetles)	45.10 ± 4.77	42.90 ± 6.47	41.20 ± 7.35	37.60 ± 3.8	1297.50 ± 360.88	1411.10 ± 315.05	1510.90 ± 366.7	764.30 ± 251.23	5077.80 ± 755.35	20.3 (4) ± 3.1	8.3 (4) ± 2.9
*A. nigrum* Schott (flies and beetles)	69.80 ± 8.04	43.30 ± 10.60	32.40 ± 7.18	29.80 ± 6.96	1779.30 ± 600.00	856.50 ± 320.68	747.50 ± 285.11	914.50 ± 206.98	990.28 ± 114.36	15.2 (5) ± 2.6	9.6 (5) ± 2.7
*A. cylindraceum* (midges)	21.60 ± 3.50	18.20 ± 1.75	18.30 ± 2.79	25.00 ± 5.81	441.30 ± 96.39	206.00 ± 39.64	332.60 ± 67.04	357.50 ± 64.01	5883.54 ± 994.40	39.7 (5) ± 7.7	16.1 (5) ± 3.9
*A. elongatum* (midges)	45.30 ± 10.90	26.10 ± 2.56	21.60 ± 3.72	16.00 ± 1.25	650.10 ± 165.71	396.50 ± 59.58	394.70 ± 74.85	507.00 ± 196.10	4656.24 ± 731.63	56.5 (2) ± 4.9	17.5 (2) ± 2.1
*A. euxinum* (midges)	17.00 ± 3.43	13.50 ± 1.65	13.50 ± 1.72	19.20 ± 4.57	248.60 ± 40.93	172.60 ± 34.56	151.70 ± 39.23	234.00 ± 57.23	8001.99 ± 653.73	76 (1)	7 (1)
*A. hygrophilum* (midges)	18.30 ± 3.02	10.60 ± 1.90	10.60 ± 2.17	8.00 ± 1.70	292.30 ± 123.08	50.50 ± 19.75	55.60 ± 15.09	69.50 ± 17.05	3367.20 ± 316.68	55.2 (5) ± 18.7	14.8 (2) ± 2.3
*A. italicum* (midges)	23.20 ± 3.19	15.20 ± 1.69	16.50 ± 1.58	31.90 ± 4.79	266.40 ± 43.79	166.00 ± 50.22	149.20 ± 42.01	425.40 ± 116.71	5619.38 ± 327.40	66.0 (5) ± 5.6	17.0 (5) ± 1.0
*A. maculatum* (midges)	29.40 ± 2.59	28.30 ± 2.71	23.60 ± 3.17	33.80 ± 3.88	441.40 ± 71.70	396.70 ± 62.50	370.70 ± 81.14	292.20 ± 87.42	9162.28 ± 804.51	69.4 (5) ± 8.8	17.2 (5) ± 2.3
*A. balansanum* (unknown)	31.80 ± 3.88	20.60 ± 5.34	34.00 ± 7.01	31.80 ± 4.83	490.40 ± 91.49	176.30 ± 42.31	1047.30 ± 294.26	391.00 ± 101.91	2873.70 ± 391.05	61.5 (2) ± 13.4	19 (2) ± 1.4
*A. besserianum* (unknown)	22.00 ± 3.71	13.10 ± 6.66	11.90 ± 2.56	32.00 ± 5.16	264.80 ± 78.30	279.10 ± 74.81	347.30 ± 72.31	416.40 ± 191.96	8449.74 ± 595.37	–	–
*A. megobrebi* (unknown)	29.90 ± 5.49	17.80 ± 2.86	0	18.70 ± 2.26	442.20 ± 82.91	502.30 ± 82.42	587.20 ± 108.28	347.60 ± 142.74	5364.05 ± 433.27	–	–
*A. purpureospathum* (unknown)	31.40 ± 8.04	27.60 ± 3.10	25.30 ± 3.95	32.20 ± 3.61	449.80 ± 114.72	365.90 ± 120.61	502.70 ± 129.50	624.20 ± 153.14	5745.39 ± 641.94	49.5 (2) ± 13.4	29 (2) ± 7.1

Number of measurements (*N*) = 10 except for the two outermost columns.

Numbers are means ± standard deviation.

S2, upper spathe chamber; S3, 1 cm above the spathe constriction; S4, central spathe blade; S5, central appendix.

**Table 4:** Differences in the structure of the slippery surfaces and area of lacunae of *Arum* spp. visited by different types of pollinators

Spathe surface	Flies and beetles	Midges	Bees	H
	(*N* = 30)	(*N* = 60)	(*N* = 20)	(d.f. = 3)
Length of papillae (μm) S2	48.63* ± 17.16	25.80† ± 10.92	20.95‡ ± 21.81	35.26 *P* < 0.001
Length of papillae (μm) S3	36.97* ± 11.47	18.65† ± 6.83	14.00‡ ± 14.59	43.26 *P* < 0.001
Length of papillae (μm) S4	32.13* ± 9.81	17.35† ± 5.15	14.95‡ ± 15.58	38.85 *P* < 0.001
Length of papillae (μm) S5	30.80* ± 7.49	22.32† ± 9.85	9.70‡ ± 10.35	35.80 *P* < 0.001
Basal area of papillae (μm^2^) Section 2	1248.80* ± 608.55	390.02† ± 170.90	786.55‡ ± 331.07	65.66 *P* < 0.001
Basal area of papillae (μm^2^) Section 3	955.33* ± 443.72	231.38† ± 134.99	822.85* ± 240.40	74.27 *P* < 0.001
Basal area of papillae (μm^2^) S4	961.27* ± 479.04	242.42† ± 141.30	1065.85* ± 457.62	79.83 *P* < 0.001
Basal area of papillae (μm^2^) S5	744.23* ± 252.96	314.27† ± 174.11	803.35* ± 220.49	62.86 *P* < 0.001
Area of lacunae (μm^2^) S2	4616.76* ± 2901.05	6115.10† ± 2072.82	0	51.75 *P* < 0.001

Numbers are means ± standard deviation and Kruskal-Wallis (H) analyses.

Mann–Whitney post hoc tests with Bonferroni correction were carried out to determine where differences lay.

Statistically distinct pairs for each plant trait are indicated with different symbols (*, †, ‡).

S2, upper spathe chamber; S3, 1 cm above the spathe constriction; S4, central spathe blade, S5, central appendix.

**Figure 3:**
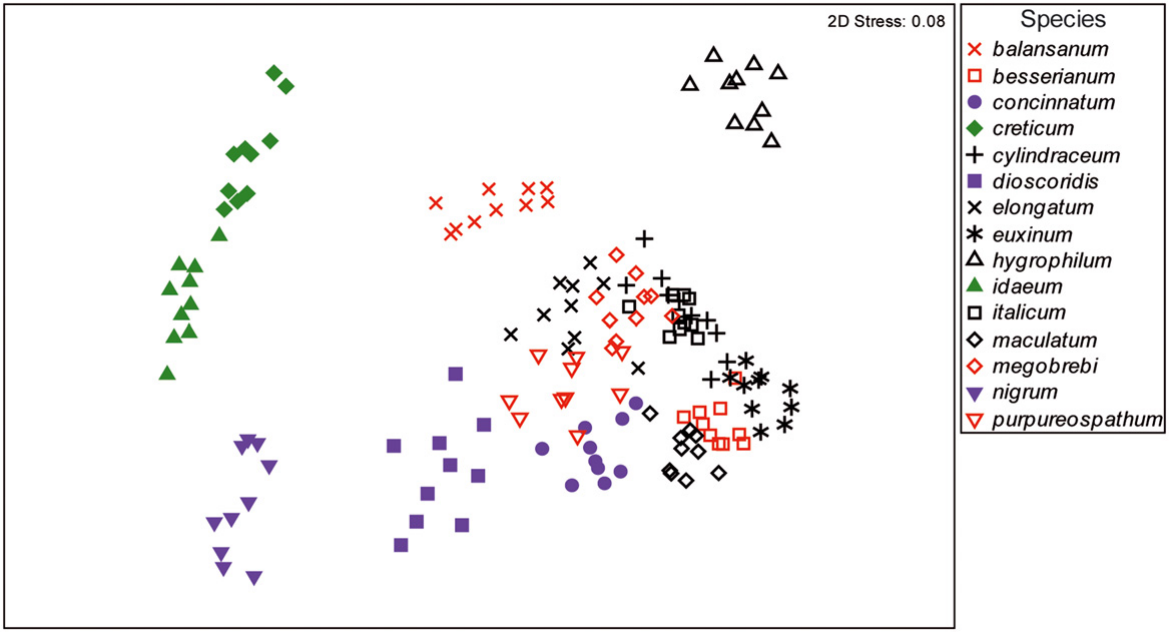
Non-metric multidimensional scaling (NMDS) of papillate cell slippery surfaces and lacunae for *Arum* spp. Species are colour-coded according to their pollinator type as follows: bees (green); flies and beetles (purple); midges (black); unknown (red).

The significant interspecific variation in the papillate cells and lacunae found in the Kruskal–Wallis tests was also evident in the NMDS and ANOSIM analyses when these features were considered in combination (Fig. 3, ANOSIM between species Global R = 0.901, *P* = 0.001). Pair-wise comparisons within the ANOSIM found significant differences between all possible species pair distributions. The largest differences between pairs of species were found between the two species pollinated by bees and each of the other species (R = 1.00, *P* < 0.001 in all cases) (Fig. 3). The largest similarity between pairs of species was found between midge-pollinated *A. cylindraceum* Gasp. and *A. megobrebi* Lobin, M.Neumann, Bogner & P.C.Boyce (pollinator unknown) (R = 0.456, *P* = 0.002) and between midge-pollinated pairs *A. cylindraceum* and *A. italicum* (R = 0.509, *P* = 0.002) and between midge-pollinated *A. euxinum* R.R.Mill and *A. besserianum* Schott (pollinator unknown) (R = 0.581, *P* = 0.004). There was some overall grouping of species according to pollinator type, with the main exception again being *A. concinnatum* (Fig. 3). Overall, bee, fly and beetle and midge-pollinated species formed clusters that were significantly different from each other. ANOSIM results if species are pooled across pollinator types (excluding species where the pollinator is unknown) give an overall significant difference across pollinator type (Global R = 0.875, *P* = 0.001). Furthermore, significant differences were found between each pair of comparisons (R = 0.919, *P* = 0.001 for bees vs. flies and beetles; R = 0.972, *P* = 0.001 for bees vs. midges; R = 0.79, *P* = 0.001 for flies and beetles vs. midges). The four species with unknown pollinator type (*A. balansanum* R.R.Mill*, A. besserianum*, *A. megobrebi* and *A. purpureospathum* P.C.Boyce) occurred among the midge-pollinated species and showed no overall significant difference to the midge-pollinated species in terms of their papillate cells and lacunae (R = 0.012, *P* = 0.340).

**Figure 4:**
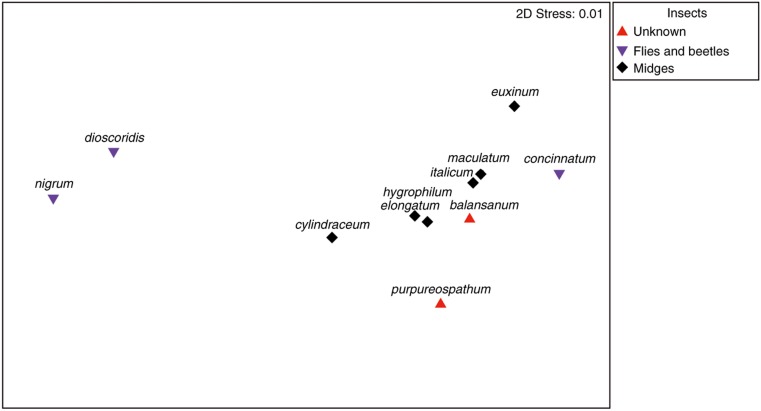
Non-metric multidimensional scaling (NMDS of data on elongated sterile flowers for *Arum* spp. Species are coded according to their pollinator type as follows: flies and beetles (triangle down); midges (square); unknown (triangle up). Note: *A. creticum* and *A. idaeum* are not displayed as they lack sterile flowers.

Strong relationships were also found between the type of insect pollinator and the number and distribution of elongated sterile flowers; generally species pollinated by bees had few or no hairs, those pollinated by flies and beetles had an intermediate number of hairs and those pollinated by midges had the most hairs (Fig. 4, ANOSIM for differences between species pollinated by flies and beetles and those pollinated by midges R = 0.59, *P* = 0.036). This result was significant despite the anomaly that *A. concinnatum* again grouped morphologically with midge-pollinated species because of its large number of elongated sterile flowers both above and below the staminate flowers.

## Discussion

Modifications in floral organs enable the adaptation to a wide array of pollinators (Claßen-Bockhoff *et al*., 2004; Whittall & Hodges, 2007), but constraints can limit the adaptability of flowers to new pollinators (Wilson *et al*., 2007; Tripp & Manos, 2008). In particular, variation in floral traits is often reduced in plants with specialized pollination because of stabilizing selection exerted by a functional group of pollinators (Fenster *et al*., 2004; Rosas-Guerrero *et al*., 2010). In our study, the trapping devices of specialized pollination traps in *Arum* showed little variation in their spatial distribution within the trap. We presume that the general design of the pollination trap is constrained because of the synorganization of different trapping devices (i.e. sterile flowers and papillate cells) required for trapping of pollinators. Nevertheless, in two closely related species, which have shifted to another functional group of pollinators (i.e. bees instead of saprophilous flies and beetles), the design of the pollination trap showed more profound modifications. Moreover, in particular papillate cells showed variation in size related to pollination by different types of insects.

### Comparison of trap design

Trap pollination by different types of insects has been recorded in different species of *Arum* (Gibernau, 2003; Quilichini *et al*., 2010). However, few studies (Knoll, 1926; Lack & Diaz, 1991) have explicitly explored the morphology and function of trapping devices that secure successful pollination. We found that the overall design of the pollination traps and the zonation of the trapping structures were uniform among *Arum* spp. studied. A uniform bauplan, with the presence of a basal inflated chamber, a narrow tube and an apical expanded section, also occurs in species of other pollination traps; for example, in *Aristolochia* L. (Aristolochiaceae) and *Ceropegia* L. (Apocynaceae) (Vogel, 1965; Oelschlägel *et al*., 2009). This uniformity is probably attributable to the common requirements for attraction, trapping and retaining of pollinators (Vogel, 1965).

Nevertheless, in *Ceropegia* and *Aristolochia* there is large variation in the size and shape of the trap (Ollerton *et al*., 2009) and the zonation and composition of trapping devices (Vogel, 1961, 1965). A major difference between *Arum* and the above-mentioned genera is that the trap in *Arum* is not a flower, but an inflorescence. In *Ceropegia* and *Aristolochia* slippery surfaces and trapping hairs (analogous to the sterile flowers in *Arum*) are formed by the epidermis of the perianth, whereas the trapping devices of *Arum* are formed by different organs (i.e. the spathe and the sterile flowers of the spadix). These organs need to be synorganized in order to ensure successful trapping of pollinators (Bröderbauer *et al*., 2012). As shown for several angiosperms, variation in floral traits is often lower in flowers with a higher degree of synorganization (Armbruster *et al*., 2009) and this might also be the reason for the conserved Bauplan and zonation of trapping devices in *Arum*.

In general, trapping hairs and slippery surfaces made up by downward-pointing papillate cells are common trapping devices that occur in several other genera of Araceae (Bröderbauer *et al*., 2012), in pollination traps of other angiosperms and even in the pitcher traps of carnivorous plants (Poppinga *et al*., 2010). Pollination traps in species of *Aristolochia* and *Ceropegia* contain either both trapping hairs and slippery surfaces or only one of the two features, and trapping hairs often occur on different parts of the floral tube (Vogel, 1961, 1965). In contrast, we found that in *Arum* spp. the slippery surfaces and the sterile flowers always occur in particular zones of the trap. This may relate to a functional difference between the trapping hairs and slippery surfaces in *Arum* and those in *Aristolochia* and *Ceropegia*. In *Ceropegia*, slippery surfaces are usually restricted to the trap entrance and ensure that insects glide into the floral chamber, whereas the subsequent arrest of insects is secured by trapping hairs that block the exit (Vogel, 1961). In contrast, sterile flowers in *Arum* do not block the entrance completely but produce slippery oil so insects cannot climb them (Knoll, 1926). Moreover, we found that in the studied *Arum* spp. papillate cells also occur below the entrance to the trap chamber and face the sterile flowers. We suppose that these slippery surfaces facilitate the retention of trapped animals that have slipped on the spathe blade and try to get past the sterile flowers by climbing the walls of the spathe chamber. Consequently, the interplay of the sterile flowers and the slippery epidermis of the spathe chamber appears to be indispensable in *Arum* in order to secure the retention of insect pollinators. This may be a major constraint for variation in the position of these trapping devices.

The *Arum* spp. that deviated most from the core design were the two closely related species *A. creticum* and *A. idaeum*. *Arum creticum* rewards pollinating bees with pollen during the staminate phase of anthesis, but has to trap the bees in the rewardless pistillate phase in order to ensure the transfer of pollen to the stigmas (Diaz & Kite, 2006). Results from the current study indicate that the switch to a trapping–rewarding pollination system coincides with trait changes that may represent a causal relationship; i.e. a reduction of lacunae and sterile flowers, but maintenance of the slippery papillae that are still necessary to make the bee glide into the lower spathe chamber containing the pistillate flowers (Diaz & Kite, 2006). The sister species, *A. idaeum*, an endemic species confined to mountain tops in Crete, also attracts bees at the lower altitude margins of its distribution range (Diaz & Kite, 2006) but is capable of autogamy by a loss of dichogamy (A. Diaz, unpubl. data). Results from the current study suggest this reduction of selective pressure for trapping may result in a concomitant reduction of all trapping devices.

The presence of trapping devices in most species studied and their uniform zonation indicates that trap pollination is a stable condition in the genus *Arum*, as it is in other aroid taxa with pollination traps (Bröderbauer *et al*., 2012). In *Aristolochia* and *Ceropegia*, switches to reward pollination appear to have occurred more often (Sakai, 2002; Ollerton *et al*., 2009), suggesting that trap pollination might be a less stable condition in these genera. However, a direct comparison between *Arum* and these two genera is difficult as both taxa are much more species-rich (*Aristolochia* > 120 species, *Ceropegia* > 180 species) and have a much wider distribution range than *Arum*. Moreover, the flowers of *Ceropegia* are not protogynous, unlike most pollination traps (Dafni, 1984; Thien *et al*., 2009), and they are highly adapted for an efficient pollen export through the presence of pollinia (Wyatt, 1978). Therefore, a switch to a non-trapping pollination syndrome may be easier in this clade.

### Relationship between pollinators and the design of trapping devices

Although different types of insects have been found to pollinate plants with floral traps (Proctor, Yeo & Lack, 1996), it is not known whether pollination traps show specific adaptations to the respective pollinator groups. We found that, in the genus *Arum*, species pollinated by different types of insects differ significantly in the size of the slippery papillate surfaces and lacunae. Although several of the taxa sharing the same pollinator type are closely related, similarities in trapping devices are unlikely to be a result of common ancestry alone, as our sample species belong to different clades of the genus and pollination syndromes in these clades have been shown to have evolved in convergence independently of the phylogenetic relationship (Linz *et al*., 2010). Therefore, we conclude that the differences in trapping devices in *Arum* are most probably attributable to adaptation to the respective pollinator types. Many studies have shown already that selection through pollinators affects floral colours, odours and shapes (Chittka *et al*., 2001; Fenster *et al*., 2004; Parachnowitsch, Raguso & Kessler, 2012). However, adaptations of the floral epidermis to the attachment organs of the insect have so far mostly been studied with respect to functional aspects (Bohn & Federle, 2004; Gaume *et al*., 2004). Our results indicate that, as for other floral organs, the design of epidermal cells is under selection by different types of pollinators, and that their role in flowers may have been underestimated. This may be particularly true for insect pollinators as they display a high diversity of attachment organs adapted to locomotion on various surfaces (Gorb, 2001).

Our NMDS analyses show that the bee-pollinated species (*A. creticum* and *A. idaeum*) form a particularly distinct cluster. A distinct clustering of bee-pollinated species was also observed for several other floral traits linked to pollination in Araceae (Gibernau, Chartier & Barabé, 2010). The differences in floral morphology are probably because of the fundamentally different behavioural and cognitive abilities and the morphological adaptations for collecting floral rewards (e.g. long tongue, corbiculae) that separate bees from saprophilous flies and beetles, which are primarily not adapted to flower visitation (Faegri & Van der Pijl, 1971). Differences in trapping features between *Arum* spp. pollinated by midges and those pollinated by flies and beetles were also apparent as the latter had larger epidermal papillae composing the slippery epidermal surfaces, generally smaller lacunae and fewer sterile flower trap hairs. The exception is *A. concinnatum*, which clustered with the midge-pollinated species. This species is reported to be visited by staphylinid beetles and various midges from different families (Urru *et al*., 2010) and was therefore coded as pollinated by flies and beetles. Nevertheless, our analyses indicate that *A. concinnatum* is morphologically more similar to midge-pollinated species. Other features such as odour and anthesis occurring at dusk (Urru *et al*., 2010; Urru, Stensmyr & Hansson, 2011), as opposed to anthesis occurring during the day in species pollinated by flies and beetles (Quilichini *et al*., 2010), are also more similar to those in midge-pollinated taxa. We postulate that the main pollinators may be midges and that the beetles may exert a low selective pressure on the inflorescences as they may visit the inflorescence only at the end of anthesis foraging for fallen pollen and thus may not be effective pollinators. By contrast, inflorescences or flowers of the same species visited by different types of effective pollinators will be under divergent selection for different floral traits (Gomez *et al*., 2008; Martén-Rodriguez *et al*., 2011). This might also be the case in the species pollinated by both beetles and flies (*A. disocoridis* and *A. nigrum*). These species are markedly different from the midge-pollinated species, but do not cluster closely together, indicating different selective pressures that are probably exerted by the different flies and beetles. Nevertheless, they show similarities in the design of their trapping devices, especially the large size of papillate cells, and in the low number of elongated sterile flowers that distinguish them from most of the midge-pollinated *Arum* spp.

Pollination by midges is the most common system in *Arum* and our results indicate a strong grouping of morphological traits for almost all midge-pollinated species. The exception is *A. hygrophilum*, which shares with other midge-pollinated species a large number of sterile flower trap hairs, but it is different in terms of the nature of its slippery surface This species has the smallest papillate cells of all taxa studied. Like other *Arum* spp., it is pollinated by midges of Psychodidae, but it differs in reportedly trapping only males instead of females (Koach, 1985). Moreover, anthesis in *A. hygrophilum* lasts up to 10 days and the midges remain trapped in the inflorescence for the whole time (Koach, 1985). In contrast, a 2-day anthesis is the standard in *Arum* (Gibernau *et al*., 2004). Whether these differences could have an impact on the nature of the slippery surfaces remains unclear.

Overall, our study shows that the trapping devices in pollination traps of *Arum* have adapted to different types of pollinators. There may be several reasons why different types of insects select for differences in the size of papillate cells forming the gliding surfaces and for different numbers of sterile flower trap hairs. In terms of the gliding surfaces, different insect pollinators have attachment organs that differ in the degree of elaboration and adaptation for climbing surfaces (Knoll, 1926; Gorb, 2001). The ability to attach to steep surfaces also depends on the body mass of the animal. The heavier the animal, the higher is the number of attachment hairs required for climbing steep surfaces (Federle *et al*., 1997; Arzt, Gorb & Spolenak, 2003). Therefore, adaptations of slippery surfaces for trapping small midges probably have to be different from those for larger and heavier flies or beetles. A large number of sterile flower trapping hairs may be useful for trapping small insects such as midges as they form a dense barrier. Finally, the various insects differ in their behaviour on flowers as flies are generally more agile than beetles (Willmer, 2011). This may also influence the way the insects are trapped best.

In conclusion, the overall design of pollination traps is rather uniform in most *Arum* spp., which is probably attributable to the synorganization of trapping devices required for trapping insects. Nevertheless, we found considerable variation in the size and number of trapping devices related to pollination by different types of pollinators. This was particularly true for differences in the construction of the slippery epidermal surfaces, a previously relatively little recognized trapping feature. Thus, the number, size and shape of the trapping devices are important variables in the reproductive ecology of floral traps. Further studies should test experimentally how changes in the size of trapping devices affect the success in trapping different types of pollinators.
